# WBP1L regulates hematopoietic stem cell function and T cell development

**DOI:** 10.3389/fimmu.2024.1421512

**Published:** 2024-11-01

**Authors:** Imtissal Krayem, Srdjan Grusanovic, Iris Duric, Nataliia Pavliuchenko, Petr Danek, Simon Borna, Jarmila Sekeresova Kralova, Tereza Skopcova, Jana Pokorna, Meritxell Alberich-Jorda, Tomas Brdicka

**Affiliations:** ^1^ Laboratory of Leukocyte Signalling, Institute of Molecular Genetics of the Czech Academy of Sciences, Prague, Czechia; ^2^ Laboratory of Hemato-Oncology, Institute of Molecular Genetics of the Czech Academy of Sciences, Prague, Czechia; ^3^ Faculty of Science, Charles University, Prague, Czechia; ^4^ Molecular Analysis of Growth Regulation in Animals, Institute of Organic Chemistry and Biochemistry of the Czech Academy of Sciences, Prague, Czechia; ^5^ Childhood Leukaemia Investigation Prague, Department of Pediatric Haematology and Oncology, 2^nd^Faculty of Medicine, University Hospital Motol, Charles University, Prague, Czechia

**Keywords:** WBP1L, transmembrane adaptor protein, hematopoiesis, hematopoietic stem cells, hematopoietic stem and progenitor cell transplantation, T cell development

## Abstract

WW domain binding protein 1-like (WBP1L), also known as outcome predictor of acute leukemia 1 (OPAL1), is a transmembrane adaptor protein, expression of which was shown to correlate with ETV6-RUNX1 translocation and favorable prognosis in childhood leukemia. It has a broad expression pattern in hematopoietic and non-hematopoietic cells. Our previous work described WBP1L as a regulator of CXCR4 signaling and hematopoiesis. Here, we show that hematopoiesis in the mice with *Wbp1l* germline deletion is dysregulated, already at the level of hematopoietic stem cells and early progenitors. We further demonstrate that thymi of WBP1L-deficient mice are significantly enlarged and contain increased numbers of thymocytes of all subsets. This can potentially be explained by increased generation of multipotent progenitors 4 (MPP4) in the bone marrow, from which the thymus-seeding progenitors are derived. We also observed increases in multiple cell types in the blood. In addition, we show that WBP1L regulates hematopoietic stem cell functionality and leukocyte progenitor proliferation and gene expression during hematopoietic stem and progenitor cell transplantation, which contribute to more efficient engraftment of WBP1L-deficient cells. WBP1L thus emerges as a regulator of hematopoietic stem and progenitor cell function, which controls leukocyte numbers at the steady state and after bone marrow transplantation.

## 1 Introduction

Leukocytes are a diverse group of cells that protect the body from harmful agents and help maintain homeostasis. They arise from hematopoietic stem cells (HSC). These cells can renew themselves, but they also have the ability to differentiate into downstream hematopoietic progenitors. The earliest progenitor stages are known as multipotent progenitors (MPP) (1). They continuously differentiate into increasingly committed progenitors, eventually giving rise to specialized leukocyte subsets. This process is accompanied by intense proliferation, allowing a relatively small number of stem cells to provide a lifetime supply of large numbers of leukocytes for the entire body (1, 2). Postnatally, hematopoiesis is mostly confined to the bone marrow with the notable exception of T cells, which undergo the largest part of their development in the thymus. However, their origin is also tied to the bone marrow, where the earliest precursors of T cells, thymus-seeding progenitors, are generated. The source of these cells is still a matter of debate. At least in part, it is within the MPP population, more specifically the MPP4 subset, also known as lymphoid-primed multipotent progenitors (LMPP). In addition, common lymphoid progenitors (CLP) are also thought to be involved (3–7). Thymus seeding progenitors migrate to the thymus and establish the population of early thymic progenitors (ETP), which then further differentiate, via multiple developmental stages, into mature T cells.

The development and production of leukocytes are tightly regulated. This control is exerted through cytokines, growth factors, extracellular matrix, and intercellular contacts, which induce intracellular signaling influencing all aspects of hematopoietic stem and progenitor cell (HSPC) function (8). Recently, we have characterized a novel regulator of signaling in the HSPCs, the transmembrane adaptor protein WBP1L (WW Domain Binding Protein 1 Like), also known as OPAL1 (Outcome Predictor of Acute Leukemia 1) (9). Initially, *WBP1L* expression was shown to correlate with favorable outcome in childhood acute lymphoblastic leukemia (10). This could potentially be explained by the association of increased levels of *WBP1L* mRNA (11) and protein (12) with *ETV6-RUNX1* (*TEL-AML1*) gene fusion in B cell precursor acute lymphoblastic leukemia, which by itself is associated with favorable prognosis (11, 13). Interestingly, a recent meta-analysis of clinical data also identified *Wbp1l* as a prognostic biomarker associated with improved progression-free survival and overall survival in serous ovarian cancer (14). However, the role of WBP1L in these neoplasms and whether it regulates processes affecting prognosis has not been investigated.

Our initial work has shown that WBP1L negatively regulates a key regulator of hematopoiesis, the chemokine receptor CXCR4, in leukocyte progenitors via the recruitment of several NEDD4 family ubiquitin ligases (9, 15). Surprisingly, our analysis of *Wbp1l^-/-^* mice revealed, that these animals were grossly normal and healthy without the major alterations in hematopoiesis that are known to occur after the disruption of CXCR4 function (9, 16). These results suggested that the hematopoietic cells were largely able to compensate for the loss of WBP1L. Interestingly, *Wbp1l^-/-^* mice showed slightly increased frequency of bone marrow LSK (Lin^-^Sca-1^+^c-Kit^+^) subset representing early HSPCs. In addition, *Wbp1l^-/-^* HSPCs engrafted better in competitive transplantations into irradiated animals (9). However, the mechanism and the types of stem and progenitor cells responsible remained unclear, as well as the contribution of *Wbp1l* to the production of most of the mature leukocyte subsets. Here, we show that function of hematopoietic stem cells and early progenitors in *Wbp1l^-/-^* mice is dysregulated. This dysregulation translates into increased thymus size, thymocyte numbers and potentially also increases in multiple cell types in the peripheral blood. We also show that WBP1L affects expression of a number of genes connected to HSPC proliferation, function and maintenance. After transplantation, this results in enhanced proliferation of early HSPCs and enhanced HSC functionality in *Wbp1l^-/-^* mice, leading to a more efficient engraftment. Our data thus reveal WBP1L as an important regulator of early hematopoiesis in the bone marrow and thymus and provide novel data on the regulation of hematopoiesis by WBP1L at steady state and after HSPC transplantation.

## 2 Results

### 2.1 Higher peripheral white blood cell counts in *Wbp1l^-/-^* mice

**Figure 1:**
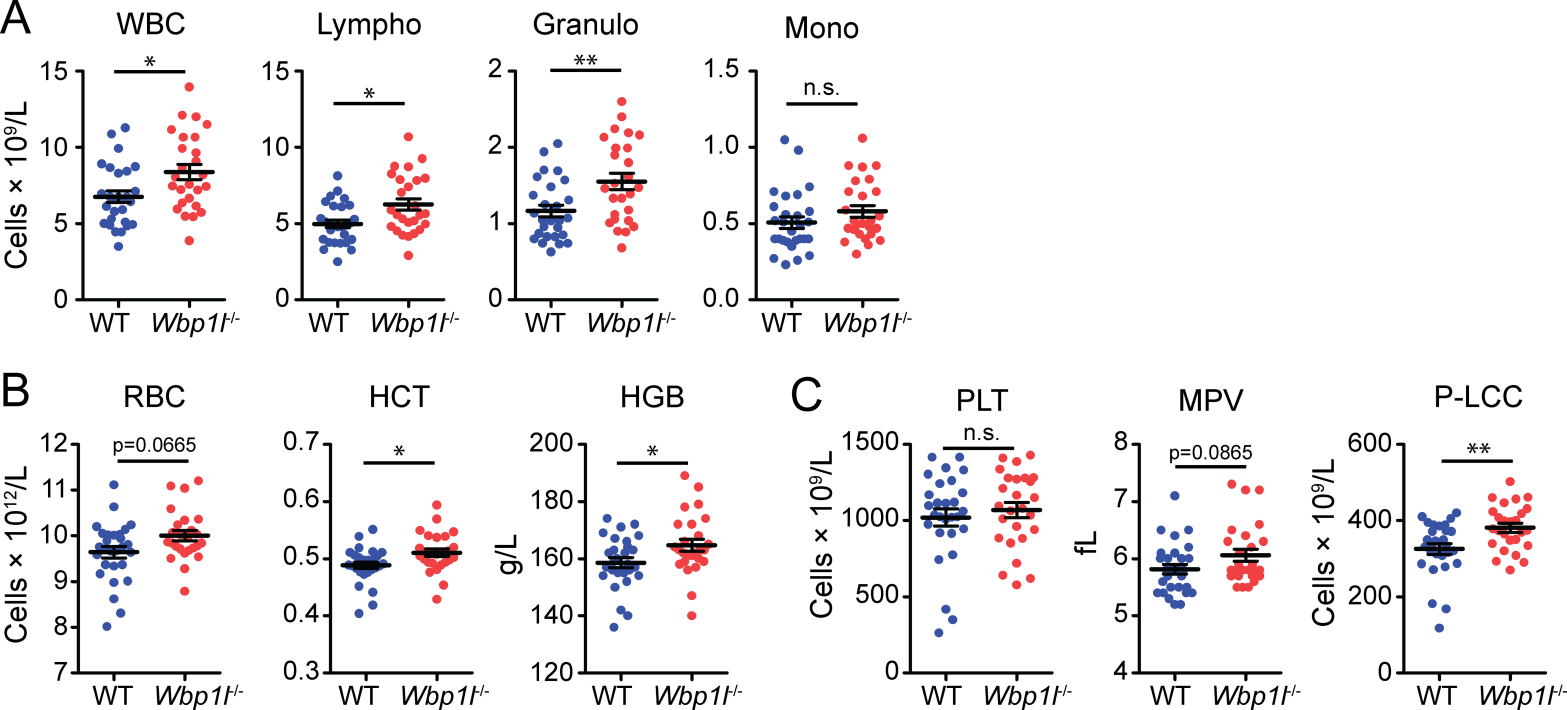
Increased numbers of blood cells in *Wbp1l^-/-^* mice. The results of blood sample analysis by hematology analyzer. **(A)** Numbers of white blood cells (WBC), lymphocytes (Lympho), granulocytes (Granulo), and monocytes (Mono). **(B)** Parameters of red blood cells. RBC, red blood cell count; HCT, hematocrit; HGB, hemoglobin concentration. **(C)** Parameters of platelets. PLT, platelet count; MPV, mean platelet volume; P-LCC, number of platelets larger than 12 fL. *p < 0.05, **p < 0.01; p-values between 0.05 and 0.1 are shown as numbers; the rest is labeled n.s. (not significant).

To assess the effect of WBP1L deficiency on blood cell composition, we measured the numbers of the main cell subsets present in the blood of *Wbp1l^-/-^* and WT mice using a hematology analyzer. Interestingly, total white blood cell count was significantly increased in *Wbp1l^-/-^* mice. The difference could also be detected at the level of individual leukocyte subsets, including lymphocytes and granulocytes (**Figure 1A**). Erythrocyte count was also slightly higher in *Wbp1l^-/-^* mice, but its increase was by a small margin outside the interval of statistical significance. Nevertheless, it was consistent with other significantly elevated parameters, namely hematocrit and blood hemoglobin (**Figure 1B**). Platelet numbers were comparable between WT and *Wbp1l^-/-^* mice. However, the proportion of large platelets (>12 fL, P-LLC parameter) was higher, likely affecting also the mean platelet volume parameter (MPV) (**Figure 1C**).

### 2.2 Enlarged thymi and increased thymocyte counts in *Wbp1l^-/-^* mice

**Figure 2:**
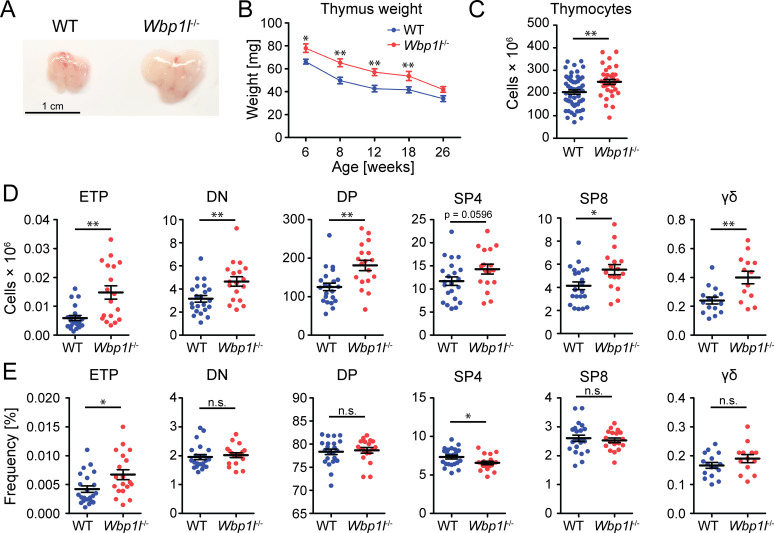
Altered thymocyte numbers in *Wbp1l^-/-^* mice. **(A)** Representative photographs of thymi from 8 weeks old WT and *Wbp1l^-/-^* mice. **(B)** Comparison of the weight of the thymi from WT and *Wbp1l^-/-^* mice of various ages. Each data point represents the average thymus weight ± SEM (N>12 mice per group per time point) **(C)** Total thymocyte numbers of 6-8 weeks old mice. **(D, E)** Numbers and frequencies, respectively, of early thymic progenitors (ETP, CD45^+^TCRβ^-^CD44^+^c-Kit^hi^), double negative thymocytes (DN, CD45^+^CD4^-^CD8^-^), double positive thymocytes (DP, CD45^+^CD4^+^CD8^+^), CD4 single positive thymocytes (SP4, CD45^+^CD4^+^TCRβ^+^), CD8 single positive thymocytes (SP8, CD45^+^CD8^+^TCRβ^+^), gamma delta T cells (γδ; CD4^-^CD8^-^CD45^+^TCRγδ^+^). For detailed gating strategy refer to the **Supplementary Figure 1**. *p < 0.05, **p < 0.01; p-values between 0.05 and 0.1 are shown as numbers; the rest is labeled n.s. (not significant).

In addition, we observed that *Wbp1l^-/-^* mice had larger thymi than WT mice (**Figure 2A**). Thymus size decreases with age in a process known as thymic involution. To test whether *Wbp1l^-/-^* mice have larger thymi because of the defect in thymic involution, we followed thymus weight in these animals until the age of 26 weeks. The weight of the thymus was higher in *Wbp1l^-/^* ^-^ animals throughout the observation period. However, it decreased with time at a similar rate as in WT mice, suggesting that thymic involution is not defective (**Figure 2B**). Thymocyte counts were also increased in *Wbp1l^-/-^* mice (**Figure 2C**). This increase could be observed for all major thymocyte subsets (**Figure 2D**, for gating strategy see **Supplementary Figure 1**). On the other hand, the frequencies of these subsets remained comparable to WT, leading to a conclusion that the increase in numbers was proportionately distributed across all thymocyte subsets (**Figure 2E**). ETP were the only cells that displayed significant increase in both, number and frequency (**Figures 2D, E**).

### 2.3 Alterations in early hematopoiesis in *Wbp1l^-/-^* mice

**Figure 3:**
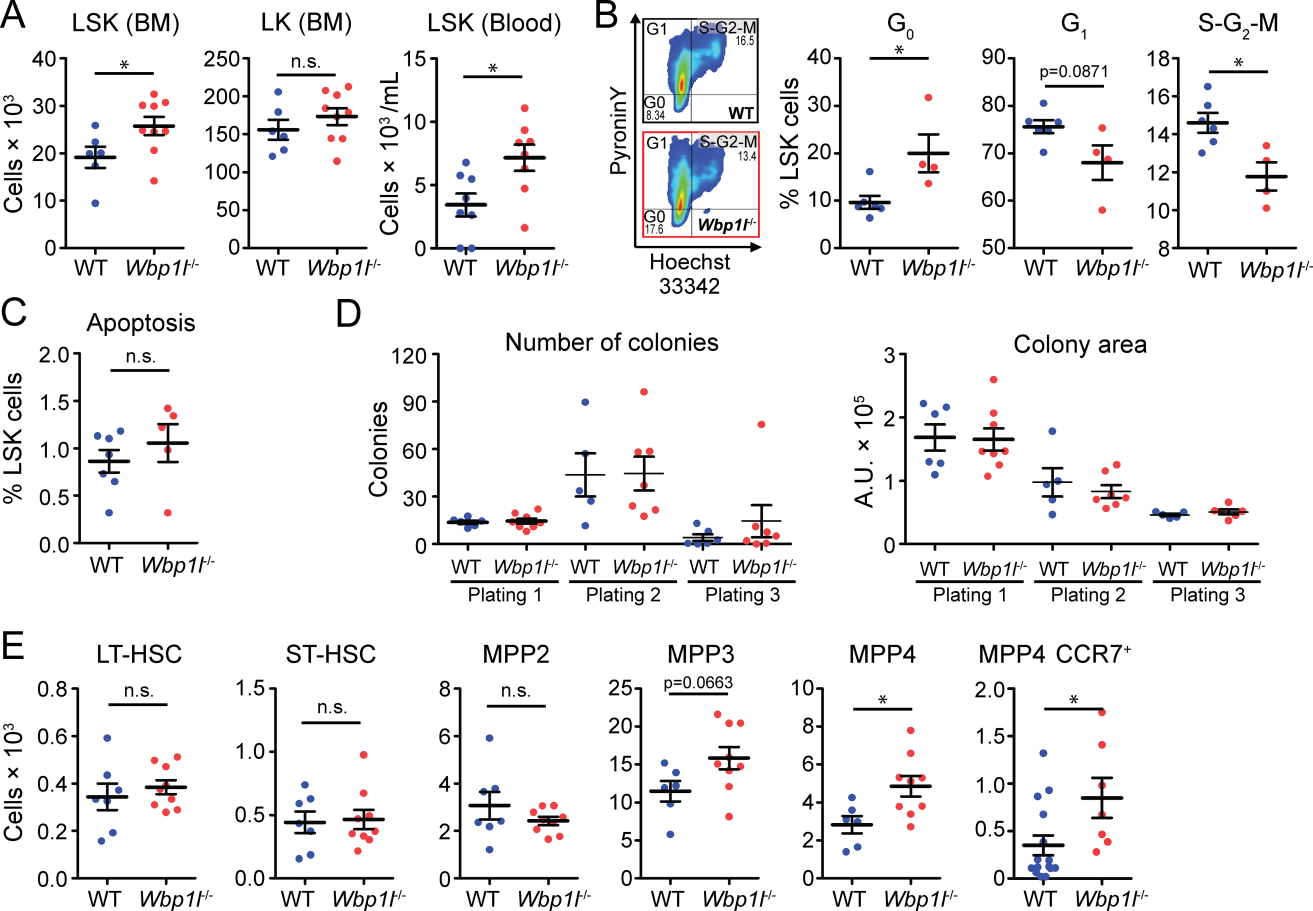
Analysis of hematopoietic progenitors from WT and *Wbp1l^-/-^* mice. **(A)** Numbers of LSK cells (Lin^-^Sca-1^+^c-Kit^+^) and LK cells (Lin^-^Sca-1^-^c-Kit^+^) per leg or per ml of blood. **(B)** Flow cytometry-based cell cycle analysis of sorted LSK cells stained with pyronin Y and Hoechst 33342 dyes. Flow cytometry plots are representative from WT (upper plot) and *Wbp1l^-/-^* (lower plot). The gates show LSK cells in G_0_, G_1_, or S-G_2_-M phases. Numbers represent percentages from parental gate. **(C)** Frequency of apoptotic LSK cells (Propidium iodide^+^Annexin V^+^) measured by flow cytometry. **(D)** Colony forming unit and replating assays of WT and *Wbp1l^-/-^* BM cells using MethoCult GF M3434 semisolid media. A total of 5 × 10^3^ BM cells was plated per well (Plating 1) in duplicates. At day 10, cells were harvested and 1 × 10^4^ of harvested cells were re-plated in a fresh media (plating 2 and 3) in duplicates. For each plating, number and size of the colonies were determined. Each dot represents the average of the duplicate pair. **(E)** Numbers of LSK cell subsets measured by flow cytometry, long-term hematopoietic stem cells (LT-HSC, CD150^+^CD48^-^), short-term HSC (ST-HSC, CD150^-^CD48^-^), multipotent progenitors (MPP2, CD150^+^CD48^+^; MPP3, CD150^-^CD48^+^; and MPP4, CD150^-^CD135^+^), and MPP4 expressing one of the thymus homing receptors CCR7 (MPP4 CCR7^+^) per leg. See here (1) for relationship to LSK subsets defined by other authors. For detailed gating strategy refer to the **Supplementary Figure S2**. *p < 0.05; p-values between 0.05 and 0.1 are shown as numbers; the rest is labeled n.s. (not significant).

ETP originate from the bone marrow precursors, which migrate to the thymus to differentiate into thymocyte subsets. In the bone marrow, at least some of these precursors are found within the population of LSK cells (Lin^-^Sca-1^+^c-Kit^+^) (3–7). These cells also give rise to all other blood cells. In our previous work, we have shown that the frequency of LSK cells was significantly increased in *Wbp1l^-/-^* mice (9). We could confirm this observation here by showing that both frequency and cell number of LSK cells in the bone marrow are significantly higher in *Wbp1l^-/-^* mice (**Figure 3A**, for gating strategy see **Supplementary Figure 2**). In contrast, the numbers of WT and *Wbp1l^-/-^* LK cells (Lin^-^Sca-1^-^c-Kit^+^), which represent progenitors downstream from LSK, were comparable (**Figure 3A**), which is also in line with our previous work (9). Interestingly, the number of LSK cells detected in the peripheral blood was also increased (**Figure 3A**). To address the mechanism behind increased LSK numbers, we analyzed the cell cycle of these cells by flow cytometry (**Figure 3B**). The data revealed that *Wbp1l^-/-^* bone marrow LSK cells are more quiescent since a larger fraction of these cells was found in G_0_ and a smaller fraction in G_1_ or S-G_2_-M phases of the cell cycle when compared to WT. Frequencies of apoptotic *Wbp1l^-/-^* and WT LSK cells were comparable (**Figure 3C**). Colony-forming unit assay did not reveal any differences between WT and *Wbp1l^-/-^* progenitors (**Figure 3D**), probably because these cultures are dominated by myeloid progenitors downstream of LSK.

Since our colony culture media do not support the growth of lymphoid cells and we have observed increased numbers of thymocytes in *Wbp1l^-/-^* mice, we decided to dissect the LSK cells into individual HSC and MPP subsets by flow cytometry (1). Interestingly, MPP4 numbers were significantly increased in *Wbp1l^-/-^* bone marrow (**Figure 3E**, for gating strategy see **Supplementary Figure 2**). The MPP4 subset, also known as LMPP (lymphoid-primed multipotent progenitors), is thought to be the source of thymus-seeding progenitors that give rise to early T-cell progenitors (1, 3–7). One of the chemokine receptors important for migration of thymus seeding progenitors to the thymus is CCR7 (17, 18). Our analysis of CCR7 expression showed that the number of MPP4 expressing CCR7 was increased in *Wbp1l^-/-^* bone marrow (**Figure 3E**). Collectively, these data suggest that the increased thymic size has roots in the dysregulation of the MPP4 progenitor subset in the bone marrow. We also observed increase in the MPP3 numbers. However, in this case, the difference was by a short distance outside the significance interval (**Figure 3E**). On the other hand, the numbers of LT-HSCs, ST-HSCs and MPP2 progenitors in *Wbp1l^-/-^* bone marrow were unchanged (**Figure 3E**).

### 2.4 Increased *Wbp1l^-/-^* hematopoietic stem cell functionality during HSPC transplantation

**Figure 4:**
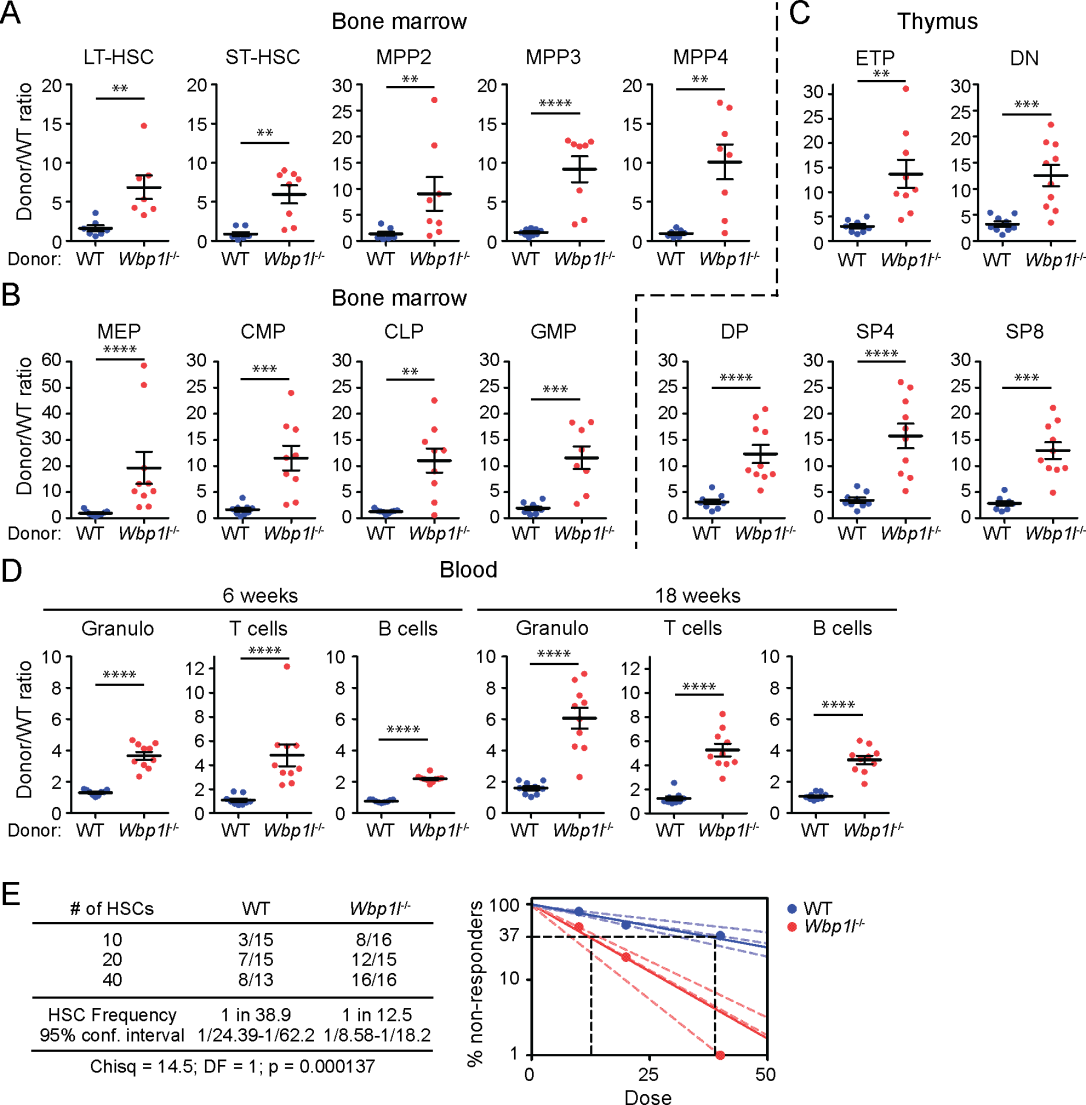
*Wbp1l^-/-^* cells outcompete WT cells in competitive bone marrow transplantations. **(A-C)** WT or *Wbp1l^-/-^* bone marrow cells (both Ly5.2^+^) were combined with equal amounts of Ly5.1^+^Ly5.2^+^ WT competitor cells and transplanted into lethally irradiated Ly5.1^+^ recipients. 18 weeks after the transplantation, the ratio of Ly5.2^+^ (WT or *Wbp1l^-/-^*) to co-transplanted Ly5.1^+^Ly5.2^+^ competitor cells (always WT) was determined for early progenitor subsets found within LSK gate **(A)**, downstream LK subsets **(B)** and thymocyte subsets **(C)**. LT-HSC, long-term hematopoietic stem cells; ST-HSC, short-term hematopoietic stem cells; MPP, multipotent progenitors; MEP, megakaryocyte erythrocyte progenitors; CMP, common myeloid progenitors; CLP, common lymphoid progenitors; GMP, granulocyte monocyte progenitors; ETP, early thymic progenitors; DN, CD4^-^/CD8^-^ double negative thymocytes; DP, CD4^+^/CD8^+^ double positive thymocytes; SP4, CD4^+^ single positive thymocytes; SP8, CD8^+^ single positive thymocytes. **(D)** The ratio of the donor (WT or *Wbp1l^-/-^*) to WT competitor cells was measured in blood samples 6 and 18 weeks after the transplantation. Granulo, granulocytes. For detailed gating strategy refer to the **Supplementary Figure S3**. **(E)** Limiting dilution transplantation assay. 10, 20, or 40 sorted LT-HSCs (WT or *Wbp1l^-/-^*) were transplanted together with 5x10^5^ WT support cells into lethally irradiated recipients. The threshold for positive engraftment was defined as >0.5% and at least two-lineage reconstitution (granulocytes, B cells, and T cells) 16 weeks post transplantation. Based on these results, frequencies of functional hematopoietic stem cells in the donor mice were determined. X‐axis indicates the number of transplanted cells. The curve fit of the log fraction of non-responding mice (solid lines) and confidence intervals (dashed lines) versus the number of mice tested is shown. The table shows the number of responders/the total number of recipients transplanted per cell dose. Frequencies of HSCs were calculated using ELDA online software based on Poisson distribution statistics (Chi‐square test; Chisq = 14.5; p = 0.000137). Data presented are from three independent experiments. **p < 0.01, ***p < 0.001, ****p < 0.0001; p-values between 0.05 and 0.1 are shown as numbers; the rest is labeled n.s. (not significant).

Increased quiescence of *Wbp1l^-/-^* LSK cells might potentially explain our previous observation that during competitive bone marrow transplantations *Wbp1l^-/-^* cells engraft more efficiently (9). Quiescent/dormant LT-HSCs, which are part of LSK subset, are known to have increased repopulation potential after transplantation (19–23). However, whether they contribute to the difference in engraftment efficiency in case of *Wbp1l^-/-^* mice has not been investigated. To address this, we performed similar competitive bone marrow transplantations as in our previous work (9). But this time we focused our analysis on hematopoietic stem cells and early progenitors, including MPPs. In addition, we also included other downstream progenitors, thymocyte subsets and blood leukocytes. We combined WT or *Wbp1l^-/-^* bone marrow cells (Ly5.2^+^) with equal numbers of Ly5.1^+^Ly5.2^+^ WT competitor cells and transplanted these mixtures into lethally irradiated Ly5.1^+^ recipients. 18 weeks after the transplantation we measured the ratio of Ly5.2^+^ (WT or *Wbp1l^-/-^*) to co-transplanted Ly5.1^+^Ly5.2^+^ WT competitors for all the cell subsets mentioned above. Strikingly, we observed a higher contribution of *Wbp1l^-/-^* cells even within the earliest developmental stages, including long-term and short-term hematopoietic stem cells and in all progenitor subsets downstream of these cells (**Figures 4A, B**). The same competitive advantage could also be observed for all thymocyte developmental stages in the thymus (**Figure 4C**). Mature leukocytes in blood also showed a similar pattern (**Figure 4D**, for gating strategy see **Supplementary Figure 3**). In addition, for each transplanted animal we collected a blood sample at 6 weeks after the transplantation, where the competitive advantage of *Wbp1l^-/-^* cells was also apparent (**Figure 4D**).

This experiment suggested increase in engraftment efficiency already at the level of *Wbp1l^-/-^* LT-HSCs. It could be explained by increased numbers of LT-HCSs or by their increased functionality. Since LT-HSC numbers in *Wbp1l^-/-^* mice measured by flow cytometry appeared normal (**Figure 3E**), we next investigated LT-HSC functionality by performing limiting dilution transplantation assay. It allowed us to measure the frequency of repopulating units, reflecting the number of functional HSCs within the LT-HSC subset defined by flow cytometry markers. We observed that WBP1L deficiency resulted in a significant, approximately 3-fold increase in functional HSCs (**Figure 4E**), demonstrating that WBP1L deficiency increases LT-HSC functionality, enhancing hematopoiesis since its earliest stages. In contrast, transplantation of purified MPP4 progenitors did not show significant differences in their engraftment efficiency until day 18, after which these cells and their progeny could no longer be detected in these mice (**Supplementary Figure 4**).

Several previous studies defined the markers of the most potent LT-HSCs. These include Sca-1, CD150 or ESAM (high expression) or c-Kit (intermediate expression). Additional markers are associated with functional bias or developmental changes of LT-HSCs such as CD41, or CD11b (20, 23–28). Our analysis of the LT-HSC subsets characterized by these markers did not reveal any alterations in *Wbp1l^-/-^* mice, with the exception of LT-HSCs expressing intermediate level of c-Kit. These cells were shown to have higher potency in transplantation assays (24). However, in *Wbp1l^-/-^* mice they were significantly reduced (**Supplementary Figure 5**). Thus, increased engraftment efficiency of LT-HSC from *Wbp1l^-/-^* mice appears to be a distinct functional property of these cells that is independent of the division into subsets characterized by these markers.

### 2.5 Changes in gene expression in *Wbp1l^-/-^* LSK cells affect their proliferation and activity

To further address the mechanism, we compared the gene expression of WT and *Wbp1l^-/-^* LSK cells after the competitive transplantation. Similar to the previous experiment, we combined Ly5.2^+^ WT or *Wbp1l^-/-^* bone marrow cells with equal numbers of Ly5.1^+^Ly5.2^+^ WT competitor cells and transplanted these mixtures into lethally irradiated Ly5.1^+^ recipients. After 5 months we isolated donor-derived Ly5.2^+^ WT or *Wbp1l^-/-^* LSK cells from the recipient mice and subjected these cells to bulk RNA sequencing.

**Figure 5:**
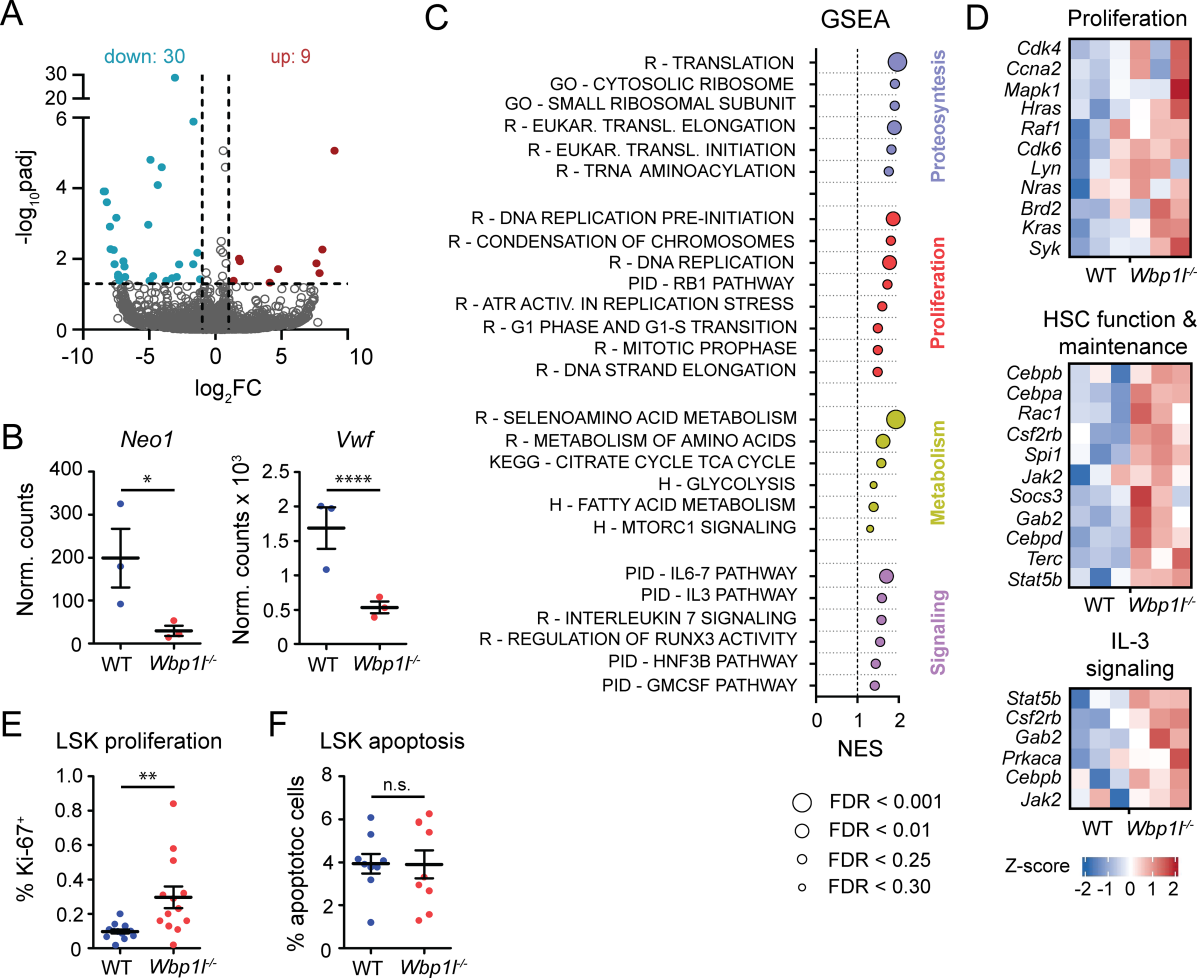
Gene expression profiling and proliferation analysis show enhanced activity of *Wbp1l^-/-^* LSK cells after competitive transplantation. **(A)** Volcano plot showing genes significantly (padj ≤ 0.05; abs[log2 FC] ≥ 1) upregulated (red) or downregulated (blue) in *Wbp1l^-/-^* LSK cells compared to WT. **(B)** Expression of *Neo1* and *Vwf* genes shown as normalized counts. Stars represent adjusted p-values calculated from RNA profiling data. **(C)** Gene set enrichment analysis of *Wbp1l^-/-^* LSK cells compared to WT. All genes were pre-ranked according to -log10(padj) and their enrichment in Reactome (R), Gene Ontology (GO), Pathway Interaction Database (PID), KEGG, and Hallmark (H) databases was calculated. Size of the points indicate FDR value. **(D)** Expression of genes involved in HSC function and maintenance, proliferation, and IL-3 signaling in WT and *Wbp1l^-/-^* LSK cells shown as Z-scores. **(E)** Frequency of proliferating (Ki-67^+^) donor-derived LSK cells 3-4 weeks after competitive transplantation. **(F)** Frequency of apoptotic donor-derived LSK cells 3-4 weeks after competitive transplantation. *p < 0.05, **p < 0.01, ****p < 0.0001; p-values between 0.05 and 0.1 are shown as numbers; the rest is labeled n.s. (not significant).

Although the expression profiles we obtained were overall relatively similar (**Supplementary Figure 6**), we identified 39 differentially expressed genes in *Wbp1l^-/-^* LSK cells (**Figure 5A**). Downregulation of Neo1 and Vwf, genes strongly involved in the biology of HSCs, is of a particular interest (**Figure 5B**). *Neo1* codes for Neogenin, a receptor on HSCs that binds niche-derived ligand Netrin1 (29, 30). Its downregulation is associated with improved HSC engraftment in competitive transplantation assays (29). Product of *Vwf* gene, von Willebrand Factor, is present in a subset of myeloid-biased HSCs, while it is absent in lymphoid-biased HSCs (see below for more detailed discussion) (31). Furthermore, gene set enrichment analysis of the whole transcriptome revealed significant enrichment of transcripts connected to proteosynthesis, proliferation, metabolism and signaling (**Figure 5C**), processes that contribute to the stem cell activity and expansion. Consistently, we observed upregulation of genes associated with proliferation, as well as HSC function and maintenance in *Wbp1l^-/-^* LSK cells. Examples of genes contributing to this result include cyclins and cyclin-dependent kinases (*Cdk4*, *Cdk6, Ccna2*), transcription factors (*Cebpa*, *Cebpb*, *Cebpd, Spi1*), and signaling molecules (*Jak2*, *Hras*, *Kras*, *Nras*, *Rac1*, *Raf1*, *Lyn*, *Syk*, *Mapk1, Csfr2b, Gab2*) (**Figure 5D**). In addition, we detected upregulation of genes connected to IL-3 signaling (**Figure 5D**), which also contributes to HSC proliferation (32).

Since the results of RNA sequencing showed increase of a number of genes associated with cell proliferation we compared proliferation of WT and *Wbp1l^-/-^* LSK cells after the competitive bone marrow transplantation. Since our data suggested that the difference in engraftment efficiency becomes apparent early after the transplantation, we analyzed the animals 3-4 weeks after the transplantation as the earliest time point when the bone marrow is sufficiently recovered to contain enough cells for analysis. Our analysis revealed significant increase in the frequencies of proliferating *Wbp1l^-/-^* LSK cells (**Figure 5E**). On the other hand, frequencies of apoptotic WT and *Wbp1l^-/-^* LSK cells were comparable (**Figure 5F**). Collectively our data suggest that increased hematopoietic stem cell functionality and increased LSK proliferation are responsible for the competitive advantage of *Wbp1l^-/-^* cells during the bone marrow transplantation experiments.

## 3 Discussion

In this work, we investigated the role of the transmembrane adaptor protein WBP1L in the regulation of hematopoiesis. Our findings reveal that its loss has significant implications for the early stages of leukocyte development, primarily affecting very early stem and progenitor cells found within the LSK gate. Numbers of these cells were elevated in *Wbp1l^-/-^* mice, while the rest of the bone marrow progenitors showed no increase. Higher leukocyte numbers were again detected in the periphery, where we observed increased blood leukocyte and thymocyte counts. In addition, *Wbp1l^-/-^* HSPCs showed advantage during competitive transplantation, resulting in substantially higher ratio of *Wbp1l^-/-^* cells to WT competitors detected in transplanted animals. This increased ratio was observed for every cell subset analyzed, both in the bone marrow and in the periphery. It was accompanied by increased proliferation of LSK cells and increased functionality of *Wbp1l^-/-^* HSCs. The changes in proliferation caused by *Wbp1l* deficiency appear to be long lasting. Increased proliferation of *Wbp1l^-/-^* LSK cells was observed 4 weeks after the transplantation and even at 5 months after the transplantation we detected increased expression of genes associated with proliferation, and functions supporting proliferation. Accordingly, we also observed increased abundance of all LSK subsets, including LT-HSCs, ST-HSCs, and all the MPPs 18 weeks after the transplantation. It is unclear for how long this increased proliferation can continue and whether it eventually results in HSPCs exhaustion.

In agreement with the data discussed above, it was previously reported that HSC proliferation intensifies after the transplantation. This increase in proliferation was maintained over the long period of time (more than 16 weeks after the transplantation), even after the hematopoiesis has been fully reconstituted (22). This transplantation-induced proliferative response is thought to be fundamentally different from steady state HSC proliferation (22, 33, 34). Our data are consistent with this notion, since *Wbp1l^-/-^* LSK cells are more quiescent than WT cells at steady state, while more proliferative, when transplanted into irradiated recipients. This observation is also in line with multiple reports showing that more quiescent hematopoietic stem cells have higher repopulation potential after transplantation (19–23). These data suggest that the effects of WBP1L deficiency on steady-state HSCs contribute to increased HSC activity after transplantation.

We bring strong evidence for increased reconstitution potential of *Wbp1l^-/-^* LT-HSC. It is less clear whether other downstream progenitors also contribute to this advantage. During the competitive transplantations, the increased ratio of donor to competitor cells apparent already at the level of *Wbp1l^-/-^* LT-HSCs (**Figure 3A**) further increased upon transition to downstream progenitors. In addition, LSK cell subset, majority of which is formed by MPPs, showed increased proliferation after transplantation. Finally, although transplantation of purified MPP4 cells did not show significant differences in engraftment, there was a trend towards increased representation of their progeny at day 18 after transplantation (**Supplementary Figure 4**). From these data, we can conclude that in addition to LT-HSC, there likely is a contribution from downstream progenitors, though it is probably not as profound.

Another interesting question is connected to the observation that there is more LSK cells in the *Wbp1l^-/-^* than in WT bone marrow, while, at the same time, they are more quiescent. It is possible that increased LSK numbers have origins earlier in the ontogenesis, perhaps even during embryonic development when the hematopoietic stem cells and early progenitors behave differently and proliferate more than in the adult mice (35, 36). They may be subject to different regulation, where WBP1L may have different role. It is also possible that there is a mild developmental block at the level of MPP4 (and potentially MPP3) in *Wbp1l^-/-^* mice, which prevents their further differentiation, resulting in their accumulation at this particular stage. Interestingly, our previous work showed reduction in the numbers of B cell progenitors in the bone marrow (9), which would be in line with this notion. However, the numbers of other progenitors downstream of MPP3 and MPP4 were not affected.

At steady state, there are other cell subsets, apart from LSK cells, that show increased numbers. In particular, these include thymocytes and peripheral blood leukocytes. Interestingly, in the *Wbp1l^-/-^* bone marrow, the most increased LSK subset were the MPP4 cells. MPP4 represent one of the populations (potentially together with CLP), from which thymus seeding progenitors are thought to be derived (3–7). Thus, it is plausible that increased numbers of MPP4 and their CCR7^+^ subset in the bone marrow then translate into increased numbers of cells migrating to the thymus, giving rise to a higher number of ETP and other thymocytes. This scenario is supported by the observation that the cell counts of most thymocyte subsets are uniformly increased so that their relative frequencies remain similar to those of the WT mice. The only cell type that shows disproportionately increased numbers are the ETPs, which show not only higher cell count but also higher frequency. These results suggest that the difference in thymocyte numbers is associated with their earliest stages and is then carried on as these cells pass through thymic development.

The molecular mechanism of how WBP1L affects hematopoietic cell numbers is at present only partially understood. We show here that the absence of WBP1L leads in transplanted LSK cells to enhanced expression of numerous genes, including genes involved in proliferation, metabolism, proteosynthesis, signaling, and HSC activity and maintenance. All these features can be linked to improved engraftment and competitive advantage of *Wbp1l^-/-^* cells. Downregulation of other genes may also contribute to this outcome. Neo1 is highly expressed on dormant HSCs, while on active HSCs its expression is downregulated. Hence, its reduced expression suggests higher activation of *Wbp1l^-/-^* HSCs. Its deficiency leads to improved engraftment in competitive HSPC transplantation (29). Another downregulated gene, Vwf, is expressed in a subset of HSCs, which show myeloid-bias, whereas the Vwf^-^ HSCs show lymphoid bias. Vwf^+^ HSCs were shown to be precursors to Vwf^-^ HSCs (31, 37). Vwf downregulation could result from increased activity of Vwf^+^ HSCs leading to generation of more Vwf^-^ HSCs. Gene expression profiles of Vwf^+^ and Vwf^-^ HSCs have been analyzed previously (37). Interestingly, Vwf^-^ HSCs also show downregulation of Neo1. However, none of the other genes differentially expressed between Vwf^+^ and Vwf^-^ HSCs were also found in our work as differentially expressed between WT and *Wbp1l^-/-^* LSK cells. Thus, the differences in the gene expression we observed likely do not indicate a change in the ratio of Vwf^+^ and Vwf^-^ HSC subsets, but rather another change in the transcription program.

How WBP1L absence leads to the changes in gene expression is not entirely clear. We have shown previously that WBP1L interacts with multiple E3 ubiquitin ligases from the Nedd4 family (9). It is thus likely that these ligases mediate its function by ubiquitinating proteins involved in the regulation of HSC gene expression, quiescence, proliferation or other processes influencing HSC functionality. Nedd4 family ligases are known to regulate a large number of proteins involved in receptor signaling, cell cycle regulation, and many other physiological processes that could influence hematopoietic cell numbers (38, 39). However, at present, it is unclear which of their many targets could be responsible for the effects caused by WBP1L deficiency. Interesting potential candidates include Notch receptors, which play a key role in T cell fate determination (6, 40, 41) or a critical hematopoietic receptor CXCR4, which we found previously to be negatively regulated by WBP1L (9). However, in all *Wbp1l^-/-^* cells we have analyzed so far, increases in CXCR4 signaling and expression were always temporary and could be only observed shortly after inducible inactivation of *Wbp1l* gene. No effects on CXCR4 signaling or expression could be detected after *Wbp1l* germline deletion (9), which was used in all the experiments in the present work. Moreover, mutations in CXCR4 enhancing its activity lead to reduced HSPC engraftment efficiency or functionality in mice and humans (42, 43) rather than increased potency observed in case of *Wbp1l* deficiency here. Thus, other targets of WBP1L and its associated ubiquitin ligases may be more important, although their identity at present remains unknown.

## 4 Materials and methods

### 4.1 Mice

*Wbp1l^fl/fl^* mice on C57BL/6J genetic background with exon 5 of *Wbp1l* gene surrounded by *LoxP* sites (derived from *Wbp1l^tm2a(EUCOMM)Hmgu^* obtained from the International Mouse Phenotyping Consortium) were described earlier (9). To establish a constitutive *Wbp1l^-/-^* knock-out strain, these mice were crossed to Cre-deleter mice Gt(ROSA)26Sor^tm1(ACTB–cre,–EGFP)Ics^ (MGI: 5285392) (44). For the competitive transplantation experiment followed by RNA profiling WBP1L deficient *Wbp1l^tm2a(EUCOMM)Hmgu^* mice on C57BL/6J genetic background were used (also labeled as *Wbp1l^-/-^*). Mice were bred in specific pathogen free conditions and maintained on a standard diet and 12 h light–dark cycle with free access to food and water at the animal facility of the Institute of Molecular Genetics. Unless otherwise indicated, mice used in the experiments were 8-12 weeks old. For transplantation assays, congenic Ly5.1 (C57BL/6J) and Ly5.1^+^Ly5.2^+^ (C57BL/6J) heterozygotes were used as recipients and competitors, respectively. Experiments in this work conducted on animals were approved by the Expert Committee on the Welfare of Experimental Animals of the Institute of Molecular Genetics and by the Czech Academy of Sciences and were in accordance with local legal requirements and ethical guidelines.

### 4.2 Phenotypic analysis of hematopoietic cells

Bone marrow single-cell suspension was prepared by crushing the femurs and tibias using a pestle and mortar. Erythrocytes were eliminated using ACK buffer (150 mM NH_4_Cl, 0.1 mM EDTA (disodium salt), 1 mM KHCO_3_). Thymocyte single-cell suspensions from 6-8 weeks old mice were prepared after homogenizing thymus tissue using syringe pistons. The cells were sterile-filtered over a 70µm sterile nylon mesh. Cells were diluted and counted either automatically (Cellometer Auto T4, Nexcelom Bioscience LLC., USA) or manually on a Bürker chamber. Cells were labeled with fluorescence-conjugated antibodies and analyzed on LSRII or Symphony flow cytometers (BD Biosciences, San Jose, CA, USA). Antibodies used for phenotypic analysis were arranged into several panels. For the analysis of the thymic cell subpopulations, two panels were used. Panel 1: CD45 AF700 (104), CD4 PerCp/Cy5.5 (GK1.5), CD8 PE/Cy7 (53-6.7), CD25 PE (PC61), CD44 APC (IM7), TCRβ FITC (H57-597), c-Kit BV605(2B8). Panel 2: CD4 PB (GK1.5), CD8α PB (53-6.7), CD45.2 PE/Cy7 (104), TCRγ/δ APC/Cy7(GL3), CD27 PerCp/efluor710 (LG.7F9), CD25 PE (PC61), CD44 BV711 (IM7)and c-Kit BV605 (2B8). Additional panels were created to analyze the hematopoietic stem cells and progenitors in the bone marrow. Panel 3: lineage cocktail mix Pacific blue [CD3 (17A2), Ly-6G(Ly-6C) (RB6-8C5), CD11b (M1/70), CD45R(B220) (RA3-6B2), Ter-119], c-Kit FITC (2B8), Sca1 BV650 (D7), CD48 PE (HM48-1), CD150 PE/Cy7 (TC15-12F12.2), CD135 APC (A2F10). Panel 4: lineage cocktail mix Pacific blue, c-Kit BV605 (2B8), sca1 APC (D7), CD34 FITC (RAM34), CD16/32 PE/Cy7 (93), IL7Ra PE (A7R34). Dead cells were stained with Hoechst 33258 (Invitrogen). For details on the individual antibodies used see **Supplementary Table 1**. Data were collected using Diva software (BD Biosciences) and analyzed with FlowJo software (BD Biosciences). For hematological analysis, peripheral blood (PB) of 7- 11 wks old WT and *Wbp1l^-/-^* mice was collected to EDTA coated tubes (078035, KABE labortechnik GmBH, Germany) and analyzed using BC-vet 30 Auto hematology analyzer (Shenzhen Mindray Animal Medical Technology Co., LTD., China).

### 4.3 Competitive bone marrow transplantation

WT or *Wbp1l^-/-^* donor strains expressed Ly5.2 (Ly5.1^-^Ly5.2^+^), competitor congenic C57BL/6J mice were WT heterozygotes expressing both Ly5.1 and Ly5.2 (Ly5.1^+^Ly5.2^+^), and congenic C57BL/6J mice used as recipients expressed Ly5.1 (Ly5.1^+^Ly5.2^-^). WT or *Wbp1l^-/-^* whole BM was isolated and mixed with competitor whole BM in a ratio of 1:1 and transplanted intravenously into lethally irradiated (6 Grey) recipients. The peripheral blood of recipient mice was analyzed every six weeks. Eighteen weeks post‐transplantation, mice were sacrificed, and the BM and the thymus were analyzed. Cells were stained with Ly5.1 and Ly5.2 antibodies to distinguish donor, competitor, and recipient cells, and with antibodies to B220, CD3, CD11b, and Gr1 to determine tri‐lineage reconstitution as previously described (45). The donor/WT ratio represents the ratio of the frequency of WT or *Wbp1l^-/-^* -derived donor cells (Ly5.2^+^) to that of competitor-derived WT cells (Ly5.1^+^Ly5.2^+^). Two donors per genotype were used. In **Figure 4**, representative experiment with one of the two donors is shown.

To analyze the proliferation and the apoptosis of donor cells, mice were sacrificed at 3-4 weeks after competitive transplantation and the BM was analyzed. Following the cell staining with antibodies to surface markers, cells were either fixed with 70% cold ethanol 1hr at -20˚C, washed and stained with ki-67 FITC (16A8) for proliferation analysis, or stained with Annexin V FITC (cat # 51-65874X, BD Bioscience) or Annexin V AF647 (cat# 640943) and Hoechst 33528 in Annexin V binding buffer (cat#422201) for apoptosis analysis. Data were collected on LSRII or Symphony flow cytometers. Two donors per genotype were used.

### 4.4 Apoptosis

In order to analyze the apoptosis of HSCs, single-cell suspensions of the bone marrow of WT or *Wbp1l^-/-^* mice were labeled with fluorescence conjugated antibodies to distinguish LSK, LK and MPP4: lineage cocktail mix, c-Kit APC (2B8), Sca‐1 BV650 (E13‐161.7), CD150 PE/Cy7 (TC15-12F12.2) and Biotinylated CD135 (A2F10), streptavidin‐APC/Cy7, followed by the annexin V FITC and propidium iodide staining in annexin V-binding buffer according to the manufacturer’s instructions.

### 4.5 Limiting dilution assay

WT or *Wbp1l^-/-^* BM HSCs were purified from Ly5.2^+^ mice as described previously (45). Briefly, the Lin^+^ fraction of the BM cells was labeled using biotinylated lineage markers: CD45/B220 (RA3‐6B2), CD3 (145‐2c11), Ter119 (TER‐119), CD11b (M1/70), and Gr1 (RB6‐8C5). These cells were further labeled with anti‐biotin magnetic beads (Miltenyi Biotec, Bergisch Gladbach, Germany) and depleted on a MACS separator (Miltenyi Biotec) according to the manufacturer’s protocol. Then, the enriched HSCs were labeled with the following antibodies: c‐Kit PE (2B8), Sca‐1 APC (D7), CD48 FITC (HM48‐1), CD150 Pe‐Cy7 (TC15‐12F12.2), and streptavidin‐eFluor450. Three doses of HSCs (10, 20 and 40 cells; Lin^−^, c-Kit^+^, Sca‐1^+^, CD48^−^, CD150^+^) were sorted on Influx instrument (BD Biosciences). Sorted HSCs were transplanted intravenously into lethally irradiated (6 Gy) mice together with 5 × 10^5^ WT BM congenic Ly5.1^+^ (C57BL/6J) support cells. 16 weeks post-transplantation, PB and bone marrow were analyzed. Ly5.1 and Ly5.2 antibodies were employed to determine donor-derived and support cells. In order to assess the tri-lineage (B‐cells, T‐cells, and myeloid cells) reconstitution, lineage‐specific antibodies (B220, CD3, CD11b, and Gr1) were employed. A recipient mouse was considered positive if the engraftment of donor cells was ≥ 0.5% Ly5.2^+^ cells contributing to at least two out of the three lineages. The frequency of HSCs was calculated with ELDA online software (46) using Poisson statistics and the method of maximum likelihood to the proportion of negative recipients in a limiting dilution setting as described previously (45, 46).

### 4.6 MPP4 transplantation

The Lin^+^ fraction of the BM cells was depleted on a MACS separator as described above in section 4.5. Then, the enriched HSCs were labeled with the following antibodies: c‐Kit PECy7 (2B8), Sca‐1 BV605 (D7), CD48 FITC (HM48‐1), CD150 PE (TC15‐12F12.2), CD135 APC (A2F10), and streptavidin‐eFluor450. 700 WT or *Wbp1l^-/-^* MPP4 cells (Lin^−^, c-Kit^+^, Sca‐1^+^, CD48^+^, CD135^+^, CD150^-^) were sorted on Influx instrument (BD Biosciences). Sorted MPP4s were transplanted intravenously into lethally irradiated (6 Gy) mice together with 5 × 10^5^ WT BM congenic Ly5.1^+^ (C57BL/6J) support cells. Blood was analyzed 8 and 18 days post-transplantation. The bone marrow was analyzed 18 days post-transplantation. Cells were stained with Ly5.1 and Ly5.2 antibodies to distinguish donor-derived cells (Ly5.2^+^) and recipient/support (Ly5.1^+^) derived cells. Four WT donors and two *Wbp1l^-/-^* donors were used. Data were collected on Symphony flow cytometer.

### 4.7 Cell cycle analysis

LSK cells were sorted similarly to the protocol described above. The HSCs were enriched by depleting Lin^+^ fraction and then labeled with the following antibodies: c‐Kit FITC (2B8), Sca‐1 APC (D7) and streptavidin‐eFluor450. Influx instrument (BD Biosciences) was employed to sort Lin^−^, c-Kit^+^, Sca‐1^+^ LSK cells. Sorted LSK cells were incubated for 20 min in phosphate-citrate buffer solution (pH 4.8) then stained with 1.5ug/ml pyronin Y and 2ug/ml Hoechst 33342. 20 000 events were acquired on Symphony flow cytometer using Diva software and analyzed with FlowJo software.

### 4.8 Colony culture assay

5x10^3^ isolated whole bone marrow cells from WT or *Wbp1l^-/-^* mice were cultured in duplicates on semi-solid media Methocult M3434 (Stemcell Technologies, Vancouver, BC, Canada) for 10 days in a 12-well plate (92412, Techno Plastic Products, Switzerland). For re-plating assays, cells were harvested, and 1x10^4^ cells were re-plated in duplicates for additional 10 days. Images from each well were acquired every 10 days using Zeiss AxioZoom.V16 macroscope with Apotome in bright field, then processed via FIJI (47) for automatic counting of colonies and estimating the colony area by a macro designed by the light microscopy core facility, IMG, Prague, Czech Republic.

### 4.9 Competitive bone marrow transplantation followed by RNA profiling

*Wbp1l^-/-^* (*Wbp1l^tm2a(EUCOMM)Hmgu^*) and wild-type BM cells from two and one donor, respectively, were isolated and mixed with Ly5.1^+^Ly5.2^+^ competitor BM cells in a ratio of 1:1, and 4 million cells were transplanted intravenously into 9-10 weeks old lethally irradiated (7 Grey) recipients. The recipient mice were sacrificed 21-22 weeks after the transplantation, and BM cells from femurs, tibias, and spines were isolated. Lin^+^ cells were depleted on an AutoMACS magnetic cell sorter (Miltenyi Biotec) using anti-biotin microbeads (Miltenyi Biotec) and biotinylated antibodies B220, CD3, Ter119, CD11b, and Gr1. The Lin^-^ cells were labeled with the following fluorophore-conjugated antibodies: c‐Kit-PE, Sca‐1-APC, Ly5.1-FITC, Ly5.2-PE-Cy7, and above listed biotinylated antibodies, followed by streptavidin‐eFluor450 staining. Lin^-^Sca1^+^c-Kit^+^Ly5.1^-^Ly5.2^+^ cells were sorted on Influx cell sorter (BD). RNA was isolated using RNeasy kit (Qiagen). Libraries were prepared with a SMARTer^®^ Stranded Total RNA-Seq – Pico Input Mammalian library preparation kit v2 (Takara) and sequenced on Illumina NextSeq 500 instrument using 75 nt single-end configuration. Reads were aligned to mm10 reference genome with the STAR algorithm and quantified with FeatureCounts function. Differential expression analysis and normalized counts were generated using DEseq2 R package. Differentially expressed genes were defined as genes with padj < 0.05 and abs[log_2_ FC] ≥ 1. For gene set enrichment analysis, all genes were pre-ranked based on their -log10(padj) and enrichment in gene sets obtained from MSigDB was calculated by GSEA software (Broad Institute) (48).

### 4.10 Statistical analysis

Differences between WT and *Wbp1l^-/-^* mice were analyzed by Mann-Whitney test using GraphPad Prism (version 5.04) software, *p-value* < 0.05 was considered significant. Although most of the data exhibit a normal distribution, this assumption could not be tested in all experiments. Therefore, to ensure consistency in the analysis of all experiments, a non-parametric test was chosen. Significant outliers were excluded based on Grubb’s Test using the GraphPad outlier calculator (https://www.graphpad.com/quickcalcs/grubbs1/). In the graphs, lines represent the mean ± SEM.; asterisks represent p values as follows *p < 0.05, ** p<0.01, ***p<0.001, ****p<0.0001; p-values between 0.05 and 0.1 are shown as numbers; the rest is labeled n.s. (not significant).

## Data Availability

The datasets presented in this study can be found in online repositories. The names of the repository/repositories and accession number(s) can be found below: E-MTAB-13908 (Array Express).
