# Supplementary material for: WBP1L regulates hematopoietic stem cell function and T cell development
Source: Front Immunol. 2024 Nov 1;15:1421512. doi: 10.3389/fimmu.2024.1421512 (PMC11563793; doi:10.3389/fimmu.2024.1421512)
Supplement: Supplementary file 1 [file DataSheet1.pdf]

Supplementary Material

1 Supplementary Figures and Tables

1.1 Supplementary Figures

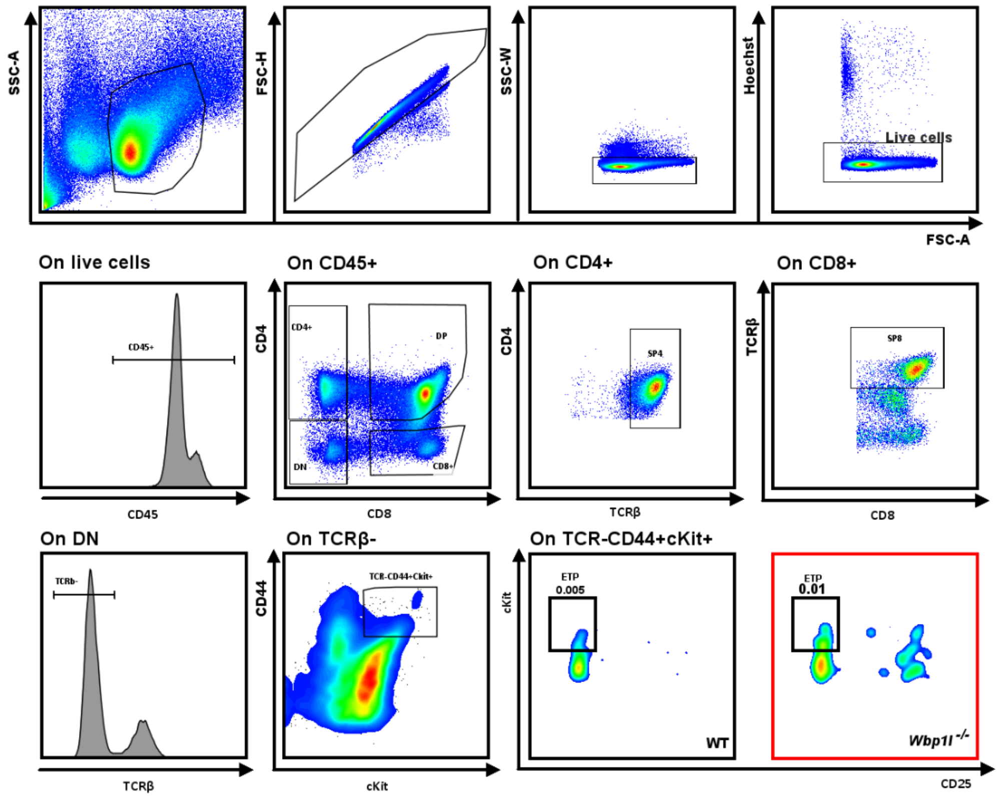

Supplementary Figure 1. Gating strategy for thymocyte subpopulations. T cells are gated from live $CD45^+$ cells. SP4 ($CD4^+TCR\beta^+$), SP8 ($CD8^+TCR\beta^+$), double positive cells DP ($CD4^+CD8^+$) and double negative cells DN ($CD4^-CD8^-$). Early thymic progenitors (ETP) are gated on the DN $TCR\beta^-$ population ($CD44^+$ c-Kit^{hi}). ETP plots from WT or *Wbp1l*$^{-/-}$ are representative from one mouse each. Values represent the frequency from total thymocytes.

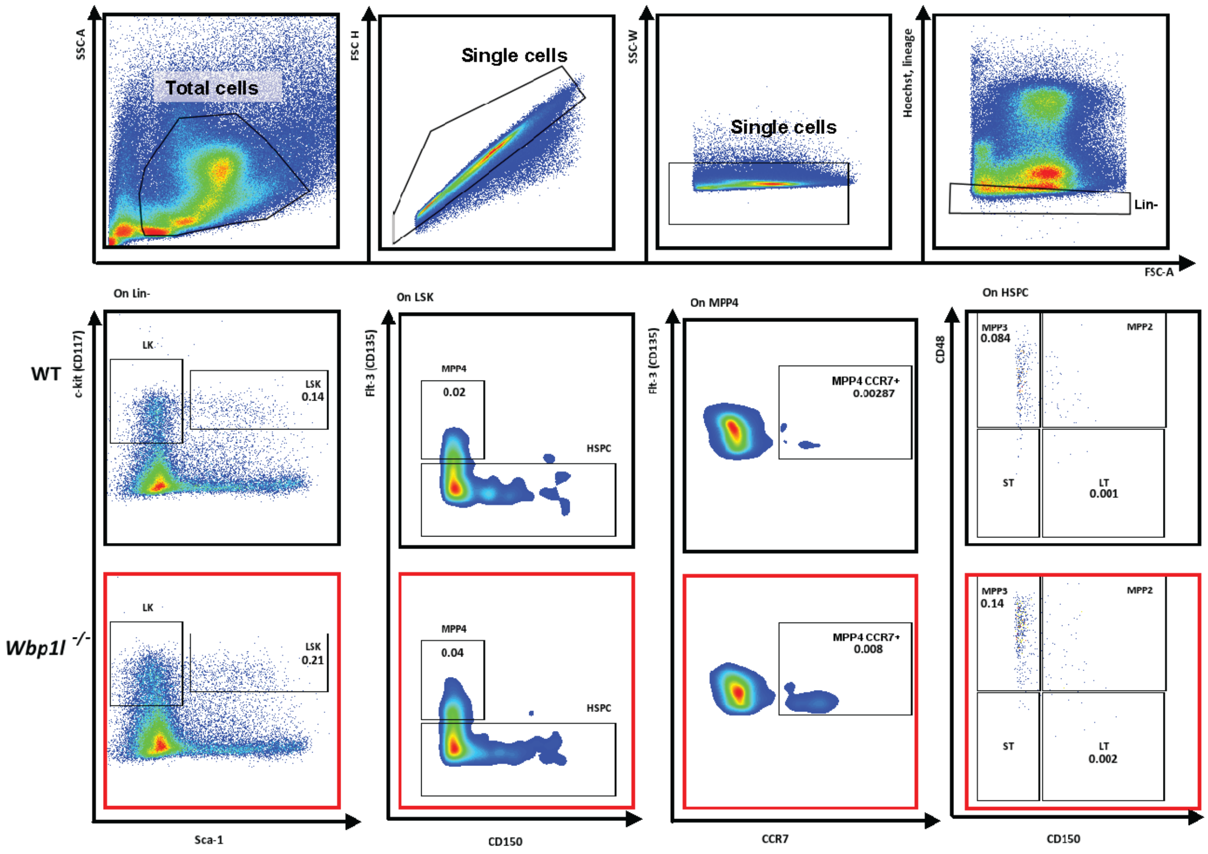

Supplementary Figure 2. Gating strategy for multipotent progenitors in the bone marrow. The multipotent progenitors are gated from live Lin$^-$ (CD3ε$^-$CD11b$^-$Gr1$^-$Ter119$^-$B220$^-$) population. LSK (Lin$^-$Sca-1$^+$c-Kit$^+$), LK (Lin$^-$Sca-1$^-$c-Kit$^+$). From LSKs, CD135$^+$ cells are the MPP4 multipotent progenitors (CD135$^+$CD150$^-$), from which MPP4 cells expressing CCR7 are derived (MPP4 CCR7$^+$). Cells lacking the expression of CD135$^-$ constitute the pool of the HSPCs, from which, MPP2 (CD48$^+$CD150$^+$), MPP3 (CD48$^+$CD150$^-$), ST-HSC (ST, CD48$^-$CD150$^+$) and LT-HSC (LT, CD48$^-$CD150$^+$) are gated. The upper (WT) and lower (*Wbp1l*$^{-/-}$) row show representative results from one mouse each. Values represent the frequency from total bone marrow cells. MPP4 CCR7$^+$ cells were analyzed independently.

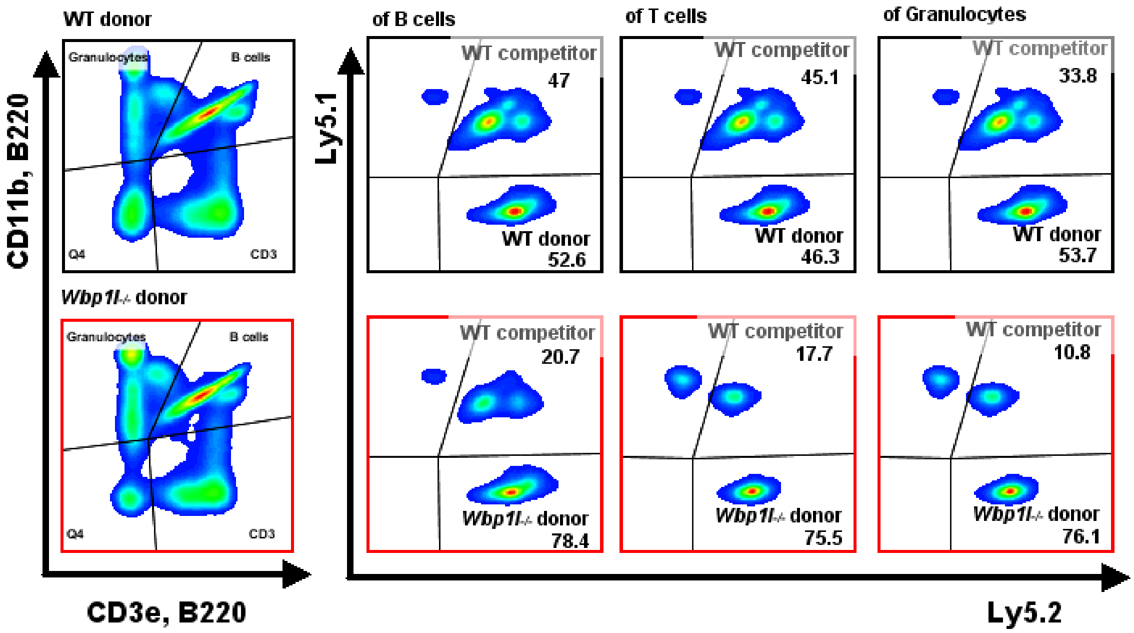

Supplementary Figure 3. Gating strategy for peripheral blood cells after competitive bone marrow transplantation. Ly5.1 and Ly5.2 markers were used to separate recipient and donor cells, respectively. WT competitors were Ly5.1$^+$Ly5.2$^+$. The upper (WT) and lower (*Wbp1l*$^{-/-}$) row show representative results from one mouse each, 18 weeks post-transplantation. Values represent the frequency from parental gate.

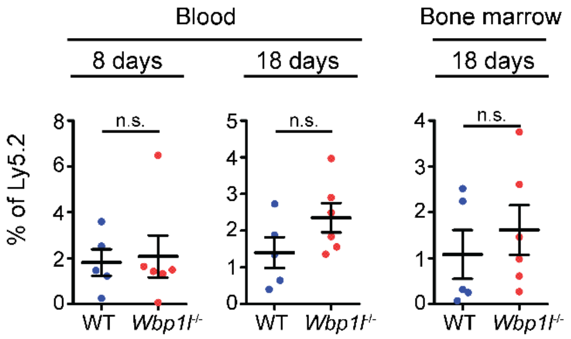

Supplementary Figure 4. Transplantation of purified MPP4. 700 sorted MPP4 (WT or *Wbp1l*$^{-/-}$, both Ly5.2$^+$) were transplanted together with 5x10^5 WT support cells (Ly5.1$^+$) into lethally irradiated recipient mice (Ly5.1$^+$). Blood was analyzed 8 and 18 days post-transplantation. The bone marrow was analyzed 18 days post-transplantation. Expression of Ly5.2 indicates the MPP4-derived cells. Each data point represents the value from one mouse. The line represents the average ±SEM, n.s. (not significant).

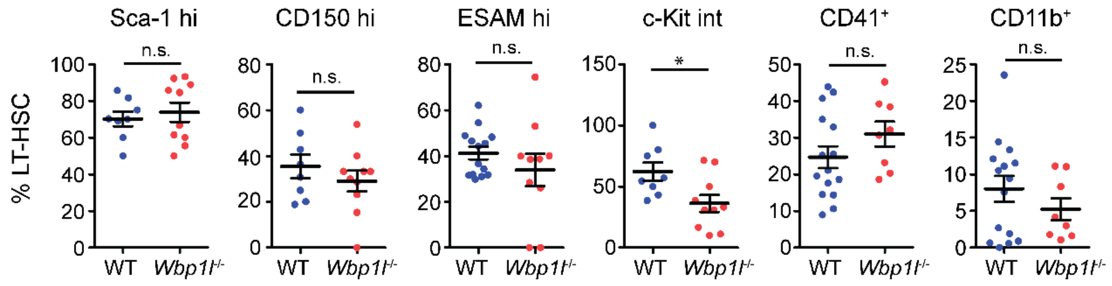

Supplementary Figure 5. Markers of LT-HSC subsets. Percentages of LT-HSC (LSK, CD150$^+$CD48$^-$) expressing (from left to right) high levels of Sca-1, CD150, ESAM, intermediate levels of c-Kit, and any level of CD41 and CD11b. For detection of CD11b, this marker was removed from the lineage cocktail. Values represent the frequency from LT-HSC. Each data point represents the value from one mouse. The line with error bars represents the average ±SEM. Asterisk represents p value $p < 0.05$; the rest is labeled n.s. (not significant).

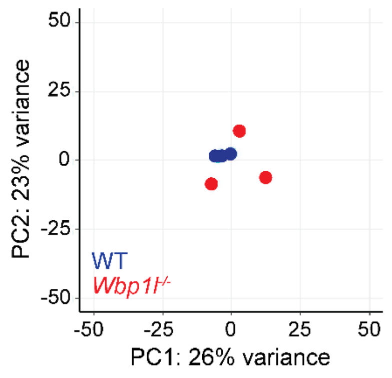

Supplementary Figure 6. Principal component analysis of RNA profiling data. The plot shows 3 WT (blue) and 3 *Wbp1l*$^{-/-}$ (red) samples.

1.2 Supplementary Tables

Supplementary Table 1. List of flow cytometry antibodies.

Antibody	Clone	Catalog number	Company
fluorescein isocyanate (FITC)-labeled anti-CD4	GK1.5	100406	BioLegend, San Diego, CA
PerCp/Cy5.5 – labeled anti-CD4	GK1.5	100434	BioLegend, San Diego, CA
phycoerythrin (PE)-labeled anti-CD8α	53-6.7	100708	BioLegend, San Diego, CA
PE/Cyanine7 anti-mouse CD8α	53-6.7	100722	BioLegend, San Diego, CA
FITC anti-mouse TCR β chain	H57-597	109206	BioLegend, San Diego, CA
allophycocyanine (APC)-labeled anti-CD3	17A2	100236	BioLegend, San Diego, CA
APC/Cyanine7 anti-mouse TCR γ/δ	GL3	118143	BioLegend, San Diego, CA
CD27 Monoclonal Antibody (LG.7F9), PerCP-eFluor™ 710	LG.7F9	46-0271-82	eBioscience™, Thermo Fisher Scientific, Inc.
PE anti-mouse CD25	PC61	102008	BioLegend, San Diego, CA
APC anti-mouse/human CD44	IM7	103012	BioLegend, San Diego, CA
Brilliant Violet 711™ anti-mouse/human CD44	IM7	103057	BioLegend, San Diego, CA
Biotin anti-mouse CD135	A2F10	135307	BioLegend, San Diego, CA
APC anti-mouse CD135	A2F10	135310	BioLegend, San Diego, CA
Brilliant Violet 785™ anti-mouse CD197 (CCR7)	4B12	120127	BioLegend, San Diego, CA

PE anti-mouse CD127 (IL-7Rα)	A7R34	135009	BioLegend, San Diego, CA
PE anti-mouse ESAM	1G8/ESAM	136203	BioLegend, San Diego, CA
APC/Cyanine7 anti-mouse CD41	MWReg30	133927	BioLegend, San Diego, CA
Brilliant violet 785™ anti-mouse/human CD11b	M1/70	101243	BioLegend, San Diego, CA
Alexa Fluor 700 (AF700)-labeled anti-CD45.2	104	109822	BioLegend, San Diego, CA
Biotin anti-mouse CD45.2	104	109804	BioLegend, San Diego, CA
Biotin anti-mouse CD8α	53-6.7	100704	BioLegend, San Diego, CA
APC anti-mouse/human CD11b	M1/70	101212	BioLegend, San Diego, CA
Pacific Blue™ anti-mouse Lineage Cocktail	17A2; RB6-8C5; RA3-6B2; Ter-119; M1/70;	133310	BioLegend, San Diego, CA
Pacific Blue™ anti-mouse CD4	GK1.5	100428	BioLegend, San Diego, CA
Pacific Blue™ anti-mouse CD8α	53-6.9	100725	BioLegend, San Diego, CA
Biotin anti-mouse Lineage Panel	145-2C11; RB6-8C5; RA3-6B2; Ter-119; M1/70;	133307	BioLegend, San Diego, CA
PerCP/Cyanine5.5 anti-mouse CD117 (c-kit)	2B8	105824	BioLegend, San Diego, CA
Brilliant Violet 605™ anti-mouse CD117 (c-Kit)	2B8	105847	BioLegend, San Diego, CA

FITC anti-mouse CD117 (c-Kit)	2B8	105806	BioLegend, San Diego, CA
Brilliant Violet 650™ anti-mouse Ly-6A/E (Sca-1)	D7	108143	BioLegend, San Diego, CA
Alexa Fluor® 700 anti-mouse Ly-6A/E (Sca-1)	D7	108142	BioLegend, San Diego, CA
Biotin anti-mouse Ly-6A/E (Sca-1)	E13-161.7	122504	BioLegend, San Diego, CA
PE anti-mouse CD48	HM48-1	103406	BioLegend, San Diego, CA
CD34 Monoclonal Antibody (RAM34), FITC	RAM34	11-0341-85	Invitrogen™, Thermo Fisher Scientific, Inc.
PE/Cyanine7 anti-mouse CD16/32	93	101318	BioLegend, San Diego, CA
PE/Cyanine7 anti-mouse CD150 (SLAM)	TC15-12F12.2	115914	BioLegend, San Diego, CA
PE anti-mouse CD127 (IL-7Rα)	A7R34	135010	BioLegend, San Diego, CA
Brilliant Violet 785™ anti-mouse CD45.1	A20	110743	BioLegend, San Diego, CA
PE/Cyanine7 anti-mouse CD45.2	104	109830	BioLegend, San Diego, CA
APC anti-mouse Ki-67	16A8	652406	BioLegend, San Diego, CA
FITC anti-mouse Ki-67	16A8	652410	BioLegend, San Diego, CA
APC/Cyanine7 Streptavidin	RUO	405208	BioLegend, San Diego, CA
Streptavidin e-Fluor 450		48-4317-82	Invitrogen™, Thermo Fisher Scientific, Inc.
